# P-1615. Inappropriate Urine Cultures: The Order Stops Here

**DOI:** 10.1093/ofid/ofae631.1782

**Published:** 2025-01-29

**Authors:** Molly Hale, Angela Christianson, Mary T Casas, Jason Cronin, Robert K Pelz

**Affiliations:** PeaceHealth, Eugene, Oregon; PeaceHealth Sacred Heart Medical Center, Springfield, Oregon; PeaceHealth, Eugene, Oregon; PeaceHealth, Eugene, Oregon; Peacehealth Medical Group, Springfield, Oregon

## Abstract

**Background:**

Our most frequently missed bundle element from catheter-associated urinary tract infection (CAUTI) case reviews was that the urine culture did not meet evidence-based criteria for testing. Possible harm to the patient from an inappropriate urine culture and subsequent treatment includes unnecessary antibiotics, diagnostic anchoring, *Clostridioides difficile* infection, and longer length of stay. Risks to the organization include artificially inflated infection rates, associated lower quality and safety scores, and reduced federal reimbursements.

**Figure 1:**
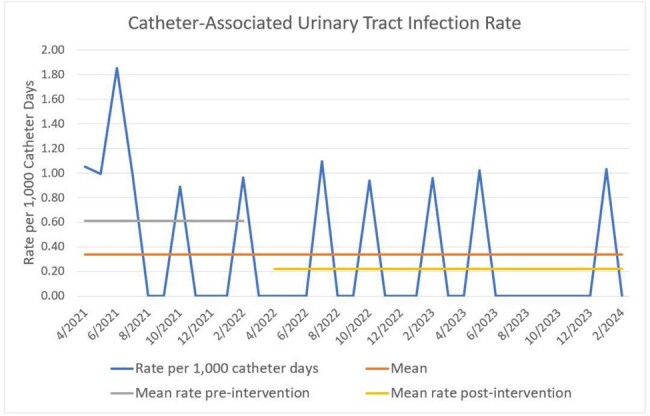
Catheter-Associated Urinary Tract Infection (CAUTI) Rate

CAUTI rate before (0.61 cases per 1,000 catheter days) and after (0.22 cases per 1,000 catheter days) diagnostic stewardship intervention in March 2022 (p=0.005).

**Methods:**

In March 2022 an automated report was built in a third-party software system using data from the electronic health record to identify urine cultures ordered in a patient hospitalized for two or more days with an indwelling urinary catheter (IUC), or who had an IUC removed in the previous two days. When a qualifying culture was identified, laboratory personnel held results and contacted the Antimicrobial Stewardship (AMS) Pharmacist. The case was discussed at AMS rounds with an Infectious Disease provider and the decision made whether to release the results. In cases where the culture was not appropriate, the patient’s provider was notified that the result would not be finalized and released. The infection rate pre- and post-intervention was calculated, and statistical analysis conducted with the Student’s T-test.

**Results:**

In the 11 months prior to implementation of the urine culture review protocol, the average CAUTI rate was 0.61 cases per 1,000 IUC days. In the 23 months since the intervention, the average CAUTI rate was 0.22 cases per 1,000 IUC days, a 63.9% reduction in the infection rate (p=0.005). 19% of orders reviewed were determined to be appropriate. The AMS team reports the volume of culture reviews is manageable. When notified that the culture results would not be released, ordering providers responded favorably to the decision and no objections or adverse patient outcomes have been observed.

**Conclusion:**

Our non-traditional intervention of enlisting the expertise of the AMS team for a diagnostic stewardship review has been successful in preventing unnecessary treatments to patients and has saved the institution from reportable CAUTI cases that did not meet clinical indication for testing.

**Disclosures:**

**All Authors**: No reported disclosures

